# Impact of extended reality on robot-assisted surgery training: a systematic review and meta-analysis

**DOI:** 10.1007/s11701-025-02559-z

**Published:** 2025-07-22

**Authors:** Michael Bickford, Fayez Alruwaili, Sara Ragab, Hanna Rothenberg, Mohammad Abedin-Nasab

**Affiliations:** 1 Rowan-Virtua School of Osteopathic Medicine, Rowan University, Stratford, NJ 08084 USA; 2 Department of Biomedical Engineering, Rowan University, Glassboro, NJ 08028 USA

**Keywords:** Robotic-assisted surgery, Extended reality, Surgical training, Simulators

## Abstract

Robot-assisted surgeries (RAS) have an extremely steep learning curve. Because of this, surgeons have created many methods to practice RAS outside the operating room. These training models usually include animal or plastic models; however, extended reality simulators have recently been introduced into surgical training programs. This systematic review and meta-analysis was conducted to determine if extended reality simulators can improve the performance of robotic novices and how their performance compares to the conventional training of surgeons on surgical robots. Using the PRISMA 2020 guidelines, a systematic review was performed searching PubMed, Embase, Web of Science, and Cochrane library for studies that compared the performance of robotic novices that received no additional training, trained with extended reality, or trained with inanimate physical simulators (conventional additional training). Articles that gauged performance using GEARS or time to complete measurements were included, while articles that did not make this comparison were excluded. A meta-analysis was performed on the 15 studies found using SPSS to compare the performance outcomes of the novices after training. Robotic novices trained with extended reality simulators showed a statistically significant improvement in time to complete (Cohen’s d = −0.95, *p* = 0.02) compared to those with no additional training. Extended reality training also showed no statistically significant difference in performance in time to complete (Cohen’s d = 0.65, *p* = 0.14) or GEARS scores (Cohen’s d = −0.093, p = 0.34) compared to robotic novices trained with conventional models. This meta-analysis seeks to determine if extended reality simulators translate complex skills to surgeons in a low-cost and low-risk environment.

## Introduction

Robot-assisted surgery (RAS) has become the standard practice for many surgical procedures, revolutionizing the field with its enhanced precision, control, and minimally invasive techniques [1, 2]. The prevalence of robotic surgery has increased over the past decade, motivated by the advances in robotic systems [3]. However, mastering the skills of robotic systems presents significant challenges due to the steep learning curve and extensive training required before being able to perform the surgeries effectively [4, 5].

RAS demands not only traditional surgical skills but also proficiency in operating complex robotic systems. Acquiring these skills involves extensive training to maneuver instruments safely and effectively [4, 5]. The unique challenges of manipulating robotic instruments through small incisions, limited or even no tactile feedback, and the need to coordinate hand–eye movements while operating through a remote interface add many layers of complexity on top of performing the surgery itself. Learning these skills solely in the operating room can be inefficient, time-consuming, and pose safety concerns for patients [4, 5]

Acquiring both technical and non-technical skills outside the operating room is now a crucial component of RAS training. Beyond traditional methods, including dry, wet, and cadaveric labs, extended reality (XR) simulators have gained significant importance in the early stages of technical skill development for RAS [6, 7]. XR simulators are safe, ethical, and repeatable alternatives that produce objective measures of performance and allow real-time feedback to trainees, often without the need for constant supervision of an experienced surgeon in a safe environment.

The emergence of XR, which encompasses virtual reality (VR), augmented reality (AR), and mixed reality (MR), offers further advancements in surgical training [8, 9]. These technologies enable trainees to practice surgical skills on a limitless number of cases with real-time feedback, thus enhancing learning outcomes and skill acquisition. Many simulators have been produced and are available in the market, giving greater access to novice surgeons. Recent literature underscores the growing importance of XR in surgical training. For instance, a 2023 study by Co et al. evaluated the XR-based systems for surgical education [9]. This study emphasizes the potential of XR to revolutionize surgical education by offering unlimited practice opportunities and real-time feedback, thereby bridging the gap between theoretical knowledge and practical skills.

Despite the growing adoption of XR-based training, no systematic review has evaluated XR training programs to other traditional methods specifically for RAS. In this systematic review, we evaluate the XR training program compared with the traditional training methods, aiming to provide insights into its effectiveness in preparing surgical trainees for the complexities of RAS. This comparison will provide valuable information on how best to integrate these emerging technologies into RAS training and improve the proficiency and safety of future surgeons.

## Method

A systematic review and meta-analysis of randomized control trials and prospective studies was conducted using the PRISMA 2020 guidelines.

### Search strategy

Four databases (PubMed, Embase, Web of Science, and Cochrane library) were searched on April 14th, 2024 using the search string (“Robot assisted Surgery” OR “Robotic Surgery” OR “Robotic Surgical”) AND (“Training” OR “Program” OR “Instruction” OR “Teach”) AND (“Virtual reality” OR “Mixed Reality” OR “Augmented Reality” OR “VR”). In every database, the articles found with this search string were exported to Rayyan.ai to go through the selection screening process.

### Study selection

**Fig. 1:**
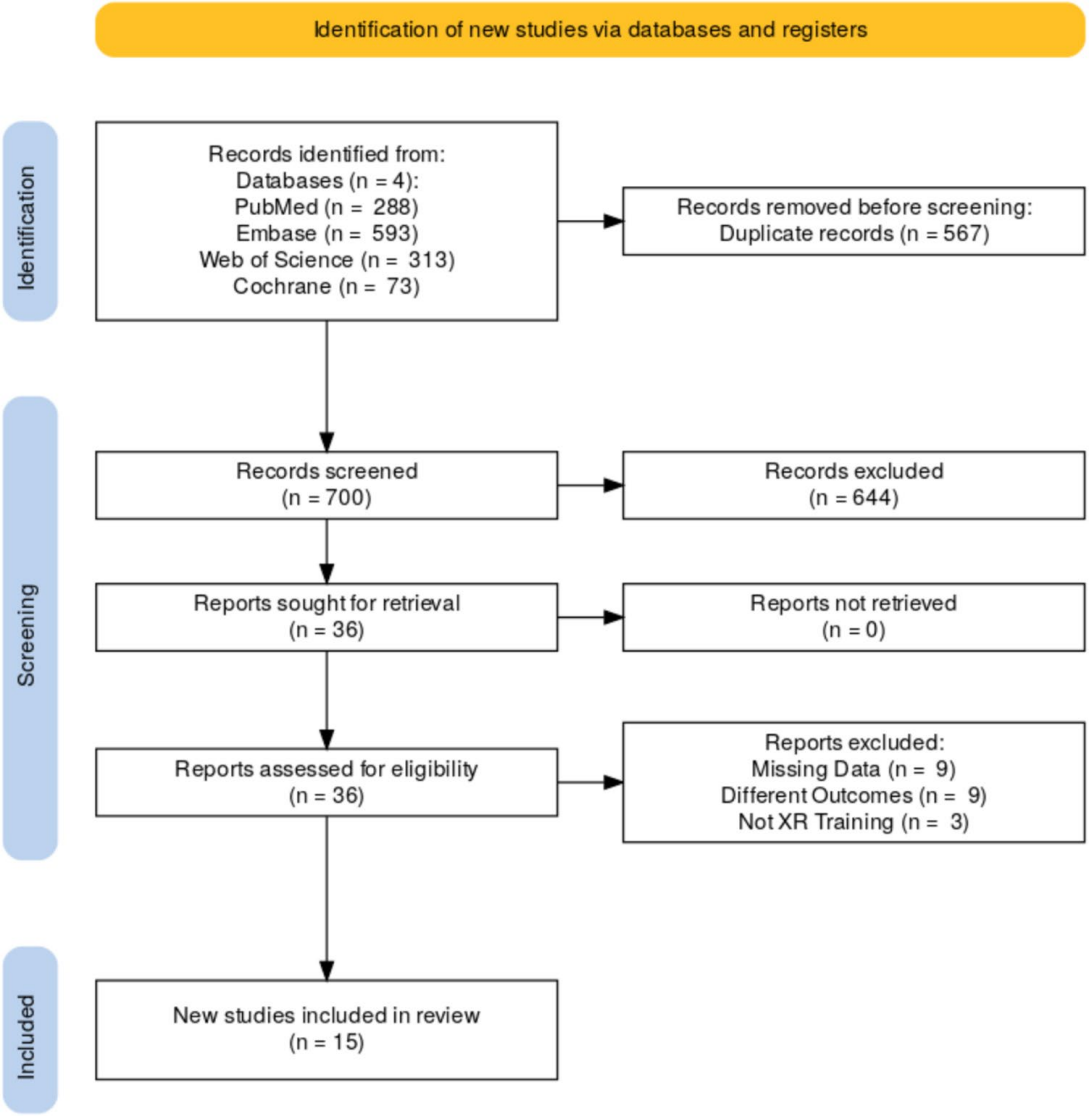
PRISMA flow diagram of the systematic review created using Rayyan.ai

The search resulted in 1267 articles that were exported to Rayyan.ai for duplication detection (Fig. 1). After screening for duplicates, 2 authors independently screened 700 articles by title and abstract based on the studies inclusion and exclusion criteria. Any disagreements between the reviewers were resolved through discussion until consensus was reached. This left 36 articles for full text review. Each article was screened independently by 2 authors and then finalized into 15 articles.

### Inclusion and exclusion criteria

Articles included in this paper were randomized-controlled trials and prospective studies that compared training programs with extended reality to either training programs with a robotic surgical device and inanimate model referred to as a dry lab, or to no additional training program after initial orientation and familiarity with the robotic surgical device. The authors included articles with participants who had never used a robotic surgical device, or robotic novices. However, level of medical education was not considered, leading to a large variation in medical training, from medical students to attending physicians. The authors also included papers that measured performance of the participants before and after the training with quantitative results like GEARS and Time to complete. Excluded articles included virtual reality validation studies where all participants used the simulator, abstracts, review articles, articles with incomplete data, and articles that looked at different parameters than GEARS or time to complete.

### Data extraction

Qualitative data were extracted from the articles after the study selection was complete. The Global Evaluative Assessment of Robotic Skills (GEARS) and time to complete were the primary outcomes observed in this paper. GEARS is a tool used to evaluate a participant’s ability to perform robotic surgical procedures [10]. Surgical performance is usually assessed by an expert surgeon in six domains related to the fundamental robotic surgical skills such as depth perception. Each domain is scored on a 5-point anchored Likert scale with a final score given as a summation of all six domains. Time to complete is a measure of how long each participant took to complete a predefined task, and in this paper, we looked specifically into suturing and knot tying with time measured or converted to seconds.

### Statistical analysis

To analyze time to complete; the mean, standard deviation, and number of participants were collected before and after the training program was completed. A meta-analysis was performed using a random-effects model on IBM SPSS Statistics for Windows, version 29 (IBM Corp., Armonk, N.Y., USA). The meta-analysis pooled the effect sizes of each article to evaluate the change in means in time to complete before and after the training program. The extent of improvement in scores was determined by the effect size of the analysis (Cohen’s d) with 95% confidence intervals. The variance between each study was calculated using a heterogeneity test using Q statistics and the I^2^ ratio (I^2^ = τ^2^/H^2^). A larger I^2^ value suggests a higher level of variance between studies. The authors compared the gear scores of participants post-training using an independent sample *t* test with a *p* value of < 0.05 determining statistical significance.

### Risk of bias

Each article was independently reviewed for risk of bias and quality of evidence by two authors. Risk of bias was determined using the Cochrane risk-of-bias template. The quality of evidence was determined using the Grading of Recommendations Assessment, Development and Evaluation (GRADE). Because this article included both Randomized control trials and prospective studies, Robbins-I was used to evaluate each article.

## Results

### Summary of findings

A total of 1267 articles were generated through electronic search of the databases listed. After deletion of duplicates, inclusion and exclusion criteria were applied and reviewed by two authors. In total, 15 randomized control trials and prospective studies were included in this study with a total of 587 participants. Each article included in the analysis is described in the summary of articles table (Table 1). 13 articles trained participants in virtual reality training, while Chowriappa et al., 2014 looked at augmented reality training. Eight of the included articles compared virtual reality training to no additional training. Eight articles compared extended reality training to dry lab training. There is an overlap with the articles Valdis et al. and Kortes et al. comparing all three categories of participants and were included in both analyses. The participants in these studies ranged from medical students to attending surgeons; however, all had limited experience when it came to robot-assisted surgeries. The two simulators used by most studies were the da Vinci Surgical Simulator (dVSS) and the Mimic dV-Trainer.

**Table 1:**
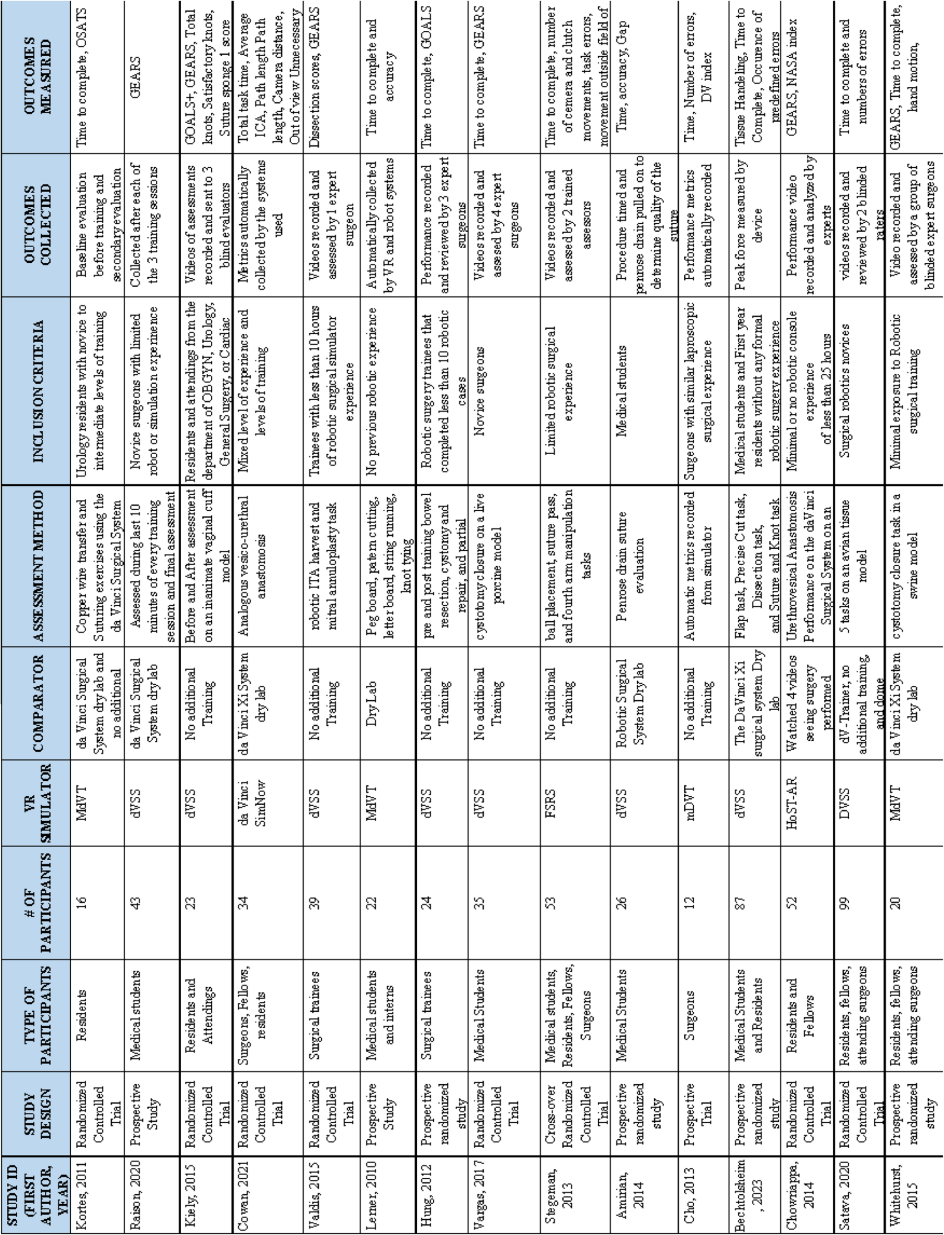
Summary table of included studies

## Summary of surgical simulators

Many surgical simulators have been developed and are available to be used to train surgeons. The most popular of these simulators is the dVSS, which is useful for training surgeons in general RAS skills, while also preparing surgeons for using the da Vinci surgical system specifically [11]. Other surgical simulators fill similar roles, or for specific styles of surgery not covered by the dVSS and are either virtual reality, augmented reality, or a hybrid of both (Table 2).

**Table 2:** Overview of extended reality simulators available

Name of simulator	Type	Type of surgery	Methods	Haptics	Cost
da Vinci Skills Simulator [11]	VR/AR	General robotic surgery	Console with access to the physical surgical robot Proficiency scores	No	$127,000 (console) $80,000 (w/o console)
dV-Trainer with Xperience Unit [12]	VR/AR	Cancerous tumor removal	Supervisor supervision w/o interruption, AR videos/interaction, Team training	Yes	$110,000
Robotix Mentor [13]	VR/AR	Hemicolectomy, Prostatectomy	VR procedures, Surgical console, Supervision console, Access to physical surgical robot	No	$137,000
Robotic surgery simulator [14]	VR/AR	Prostatectom, Hysterectomy	Video Modules, Force feedback, 3D viewer with monitor	Yes	$120,000
ProMIS simulator [15]	Hybrid	Laparoscopic surgery	Laparoscopic Tools, Tactile Tasks, Objective proficiency scores	Yes	$50,000
Simsurgery educational platform [16, 17]	VR	Laparoscopic surgery	General surgical robot simulator, Time-sensitive tasks	No	$62,000
Robossis surgical simulator [18]	VR	Femur fractures	Robossis system femur fracture alignment	Yes	NA
Microsoft hololens simulation [19]	MR	Open liver surgery, Laparoscopic surgeries	Microsoft HoloLens 2, Unity AR Software, da Vinci Research Kit (DVRK)	No	$3,500 (HoloLens 2), $250 (DVRK), $180/Month
Simendo [20]	VR-based	Urology	Robotic urology, laparoscopic & robotic techniques	No	NA
Senhance simulator [21]	VR-based	General robotic surgery	Senhance system procedures, haptic feedback, and eye-tracking	Yes	NA
Versius trainer [22]	VR-based	General robotic surgery	Designed for the Versus robotic system, general surgery, and specialties	Yes	NA
FlexVR by mimic technologies [23]	VR-based	Flexible robotic surgery (i.e., NOTES)	Flexible robotic system simulation with procedural walkthroughs	No	$1,995 /Month
NeuroTouch [24]	VR-based	Robotic Neurosurgery	VR with tactile feedback, robotic brain surgery simulation	Yes	NA

*NA* Not Available

### XR vs. no additional training

To determine if extended reality is an effective form of training surgeons. We compared participants that were trained in extended reality to participants that did not receive any additional training outside of being introduced to the mechanics of the surgical robot. Time to complete and GEARS score were used to compare the two groups. These measurements were recorded after both groups completed their forms of training.

**Fig. 2:**
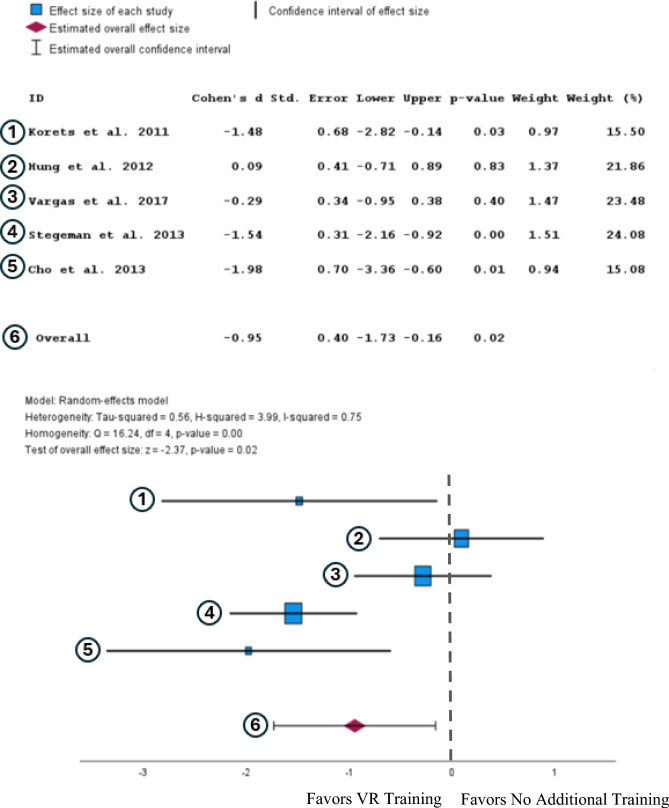
Forest plot comparing the effect size of VR training to no additional training on time to complete after completing a training regimen. [25–29]

A random-effects analysis was used for this meta-analysis and showed that participants with virtual reality training took statistically significant less time to complete tasks than participants with no additional training (Cohen’s d = −0.95, 95% CI [−1.73, −0.16], *p* = 0.02) (Fig. 2).

**Fig. 3:**
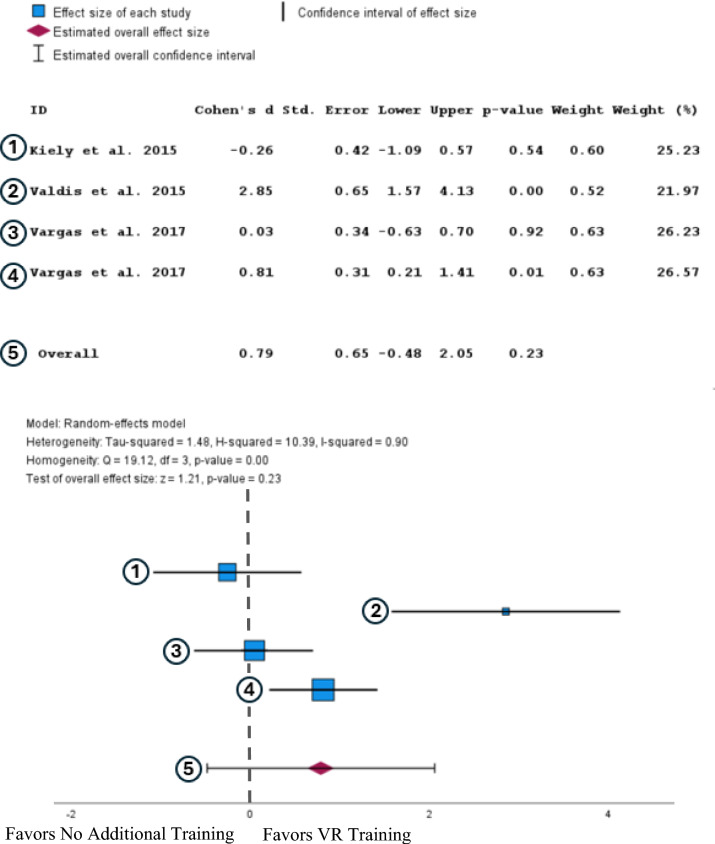
Forest plot comparing the effect size of VR training to no additional training on GEARS scores after completing a training regimen. [27, 30, 31]

A random-effects analysis of the GEARS scores showed that participants with extended reality training had higher GEARS scores than participants without any additional training. With the training having a large effect on the scores (Cohen’s d = 0.75, 95% CI [−0.48, 2.05]). However, this result is not statistically significant (*P* = 0.23) (Fig. 3).

### XR vs. dry lab

To determine how extended reality training compares to traditional styles of RAS training, the authors compared participants that were trained with extended reality simulators to participants that used inanimate, dry lab simulators.

**Fig. 4:**
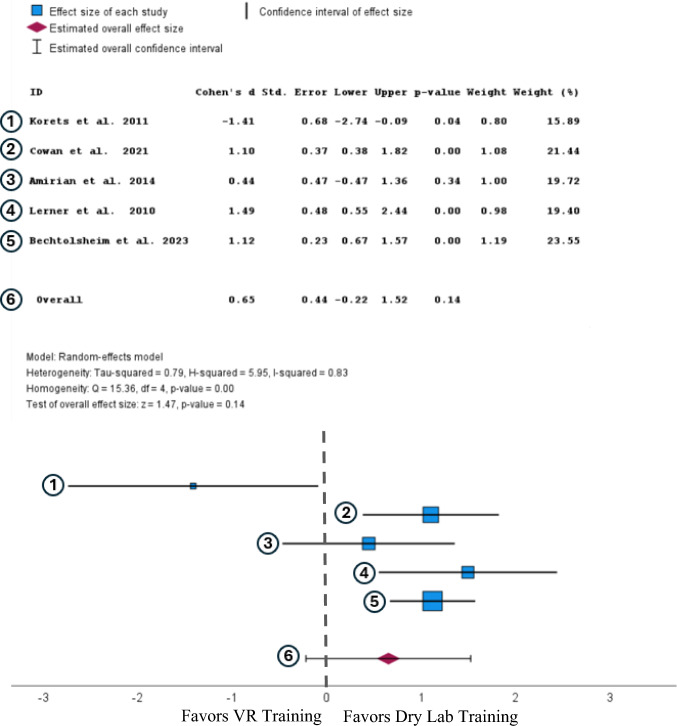
Forest plot comparing the effect size of VR training to dry lab training on time to complete after completing a training regimen. [25, 32–35]

A random-effects analysis of time to complete showed a moderate, not statistically significant increase in time to complete for participants trained in virtual reality compared to participants trained in a dry lab (Cohen’s d = 0.65, 95% CI [−0.22,1.52], *p* = 0.14) (Fig. 4).

**Fig. 5:**
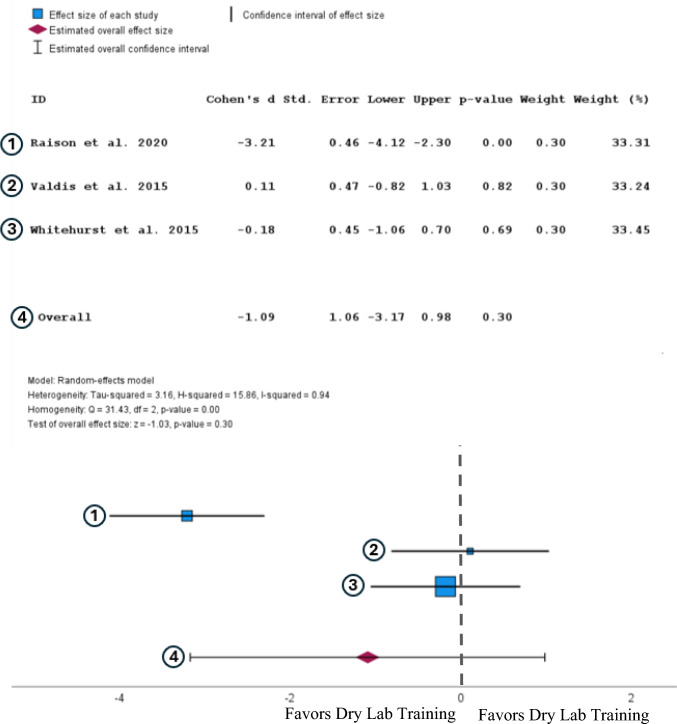
Forest plot comparing the effect size of VR training to dry lab training on GEARS Score after completing a training regimen. [31, 36, 37]

A random-effects analysis of the GEARS scores showed that participants with extended reality had lower GEARS scores than those that were trained in extended reality. (Cohen’s d = −1.09, 95% CI [−3.17, 0.98]). However, this result is not statistically significant (*P* = 0.30) (Fig. 5).

### GEARS score t test

On top of the random-effects analysis, an independent samples t test was done to compare the mean GEARS score between VR and No Training groups (Table 3). This is because each group satisfied conditions for an independent samples *t* test [11], as the studies were large in number (63 vs. 60 participants), worked in independent environments, and had highly homogenous standard deviations. The independent samples t test yielded an effect size strongly in favor of the VR trained group; 0.964 (95% CI 0.589 to 1.336). The threshold for a large effect size is 0.8 [13]. The mean GEARS score also crudely favored the VR trained group at 16.73, compared to no training at 13.91. An independent samples t test was also used to compare the mean GEARS score between VR and Dry Lab groups. Each group satisfied conditions for an independent samples t test [11], as they were large in number (44 vs. 37 participants), worked in independent environments, and had highly homogenous standard deviations. The independent samples t test yielded an inconclusive effect size of −0.093. This effect size rests in a 95% confidence interval of −0.530 to 0.345.

**Table 3:** Summary of independent sample *t* tests

Group	Mean ± SD XR	Mean ± SD Dry lab training	Difference in means		Cohen’s d	*P* value
XR vs. No Training	16.73 ± 2.8, *n* = 63	13.91 ± 2.95, *n* = 60	2.82 ± 0.53 [95% CI 1.78, 3.86]		0.964 [95% CI 0.589, 1.336]	< 0.001
XR vs. Dry lab	13.43 ± 6.7, *n* = 44	14.08 ± 7.33, *n* = 37	−0.65 ± 1.56	−0.093 [95% CI −0.530, 0.345]		0.34

### Risk of bias and GRADE analysis

**Fig. 6:**
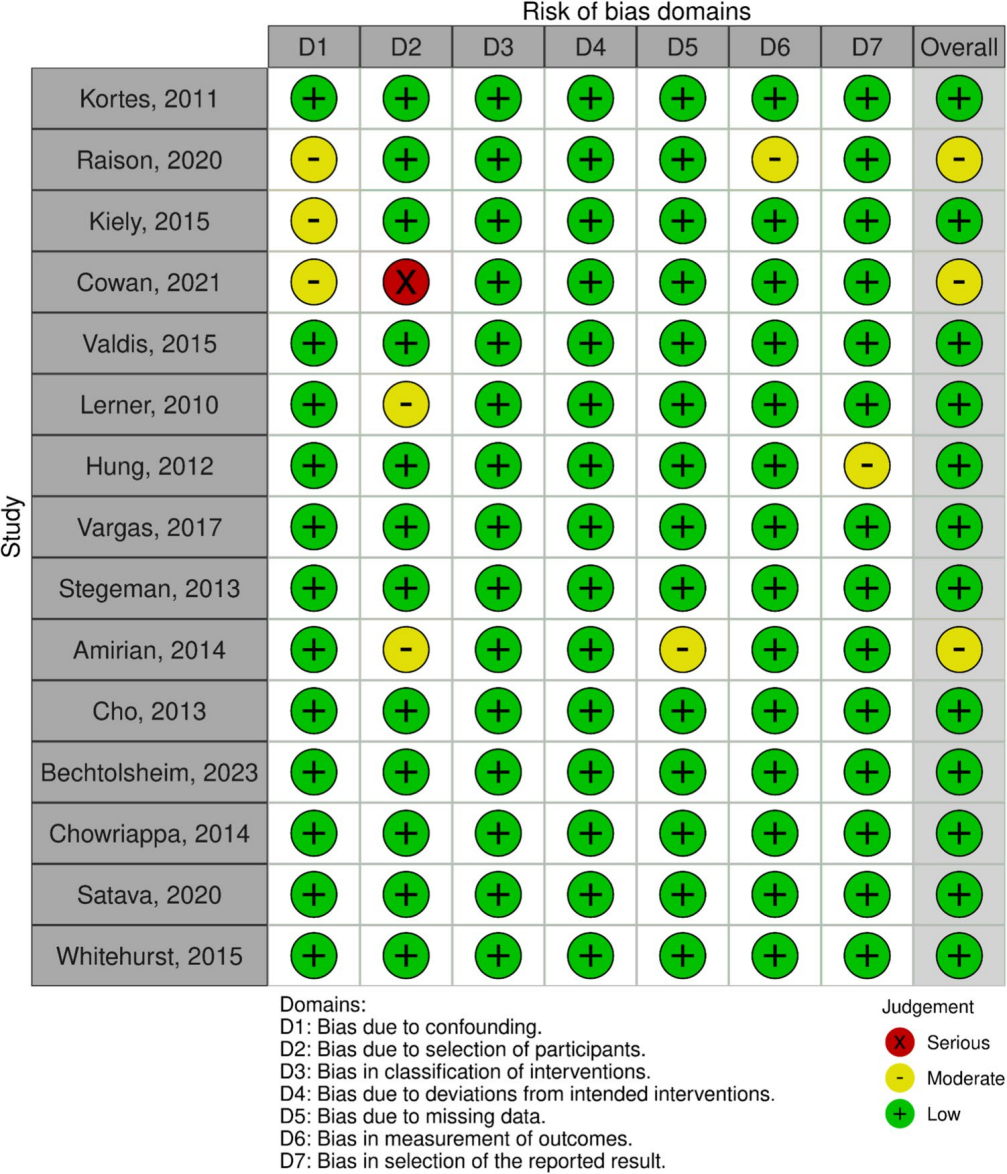
Traffic light plot portraying the Robbins-I assessment

**Fig. 7:**
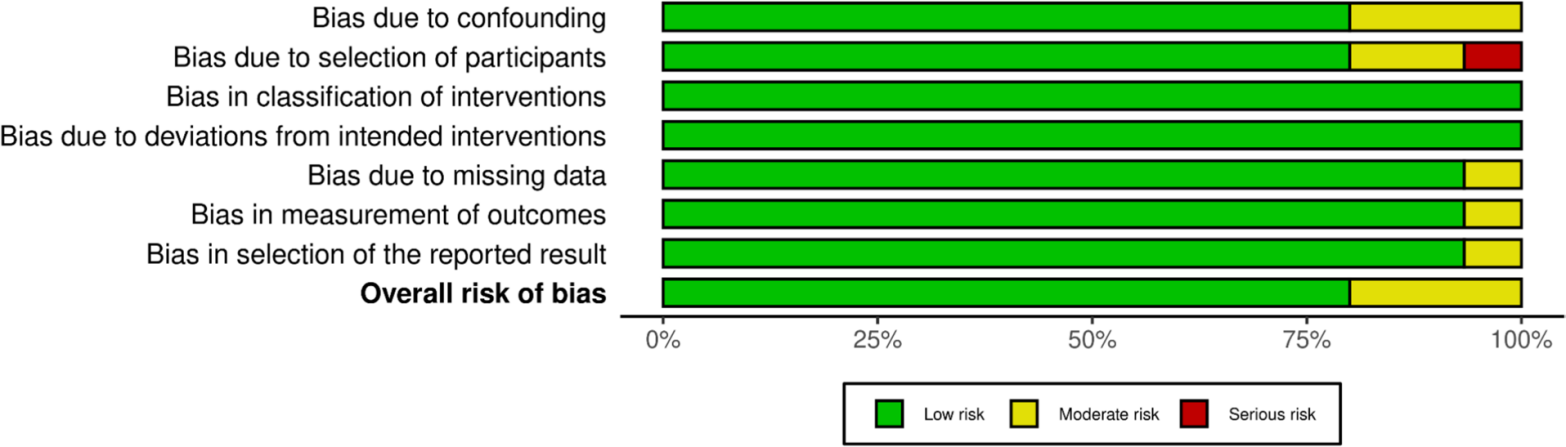
Robbin I summary plot

**Fig. 8:**
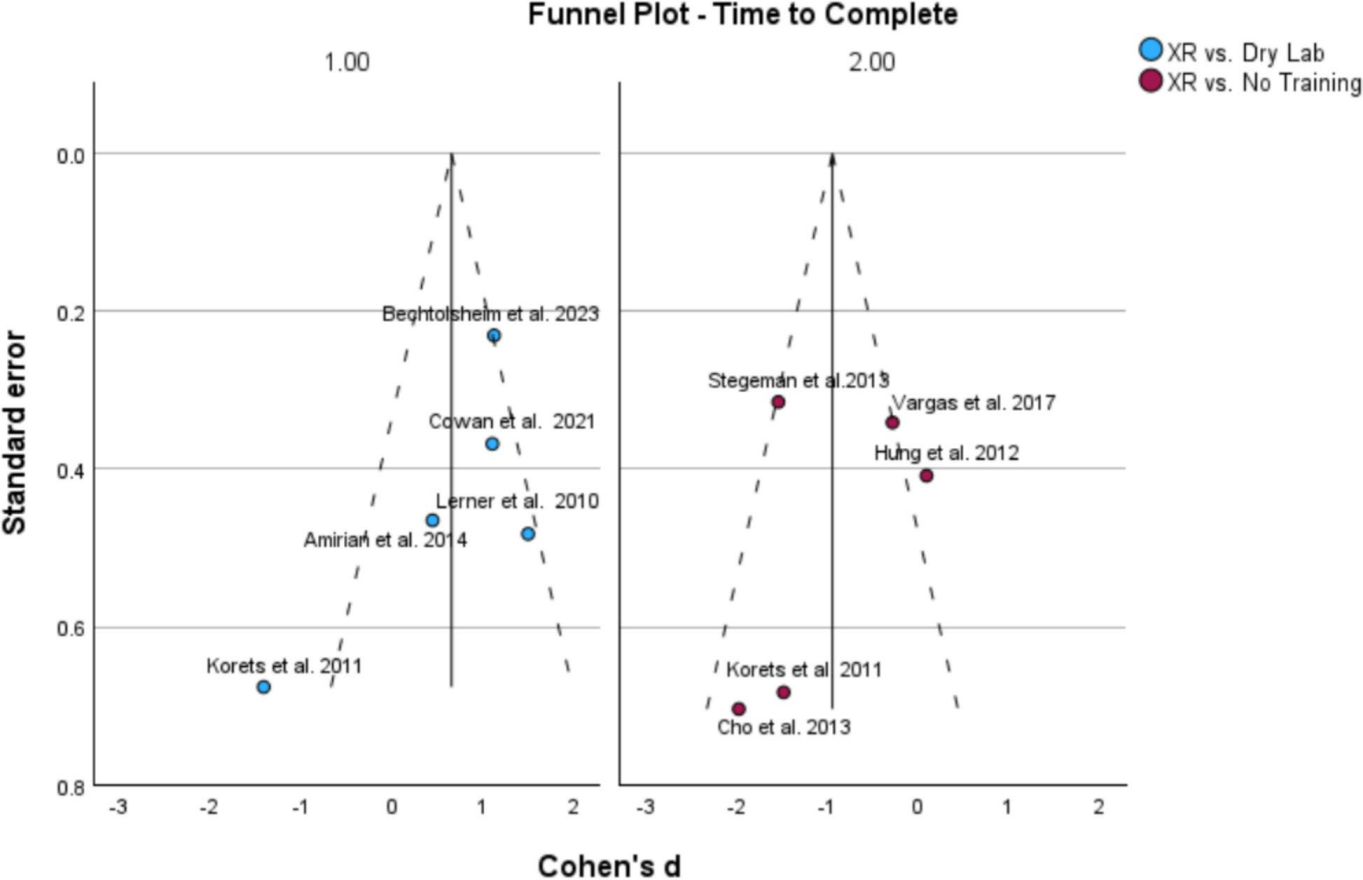
Funnel plot displaying the standard error of each study’s main effect size that compares the GEARS scores of participants

Each study was evaluated for an overall risk of bias using Cochrane’s Risk of Bias handbook and tool by two authors independently [14]. A Robbins-I analysis was used due to the articles in this study including randomized-controlled trials and prospective trials. Twelve of the articles were found to have a low risk of bias, while three of the articles were found to have a moderate risk of bias. Each domain of Robbins-I is represented in Figs. 6, 7, and 8.

To determine the certainty of the evidence in this study, two independent authors performed a GRADE analysis. The analysis revealed the studies in this review are a moderate level of evidence (Table 4).

**Table 4:** Overall GRADE analysis of included articles

Author	Year	Study design	Intervention	Risk of bias	Inconsistency	Indirectness	Imprecision	Grade
Korets et al [25]	2011	Randomized-controlled trial	MdVT	Not Serious	Serious	Not serious	Serious	Moderate
Raison et al [36]	2020	Prospective study	dVSS	Serious	Serious	Not serious	Not serious	Moderate
Kiely et al [30]	2015	Randomized-controlled trial	dVSS	Not Serious	Serious	Not serious	Not serious	Moderate
Cowan et al [32]	2021	Randomized-controlled trial	da Vinci SimNow	Serious	Serious	Not serious	Not serious	Moderate
Valdis et al [31]	2015	Randomized-controlled trial	dVSS	Not Serious	Serious	Not serious	Not serious	Moderate
Lerner et al. [34]	2010	Prospective Study	MdVT	Not Serious	Serious	Not serious	Not serious	Moderate
Hung et al [26]	2012	Prospective randomized study	dVSS	Not Serious	Serious	Not serious	Not serious	Moderate
Vargas et al [27]	2017	Randomized-controlled trial	dVSS	Not Serious	Serious	Serious	Not serious	Moderate
Stegeman et al [28]	2013	Cross-over randomized-controlled trial	FSRS	Not Serious	Serious	Not serious	Not serious	Moderate
Amirian et al [33]	2014	Prospective randomized study	dVSS	Serious	Serious	Not serious	Not serious	Moderate
Cho et al [29]	2013	Prospective trial	MdVT	Serious	Serious	Not serious	Serious	Moderate
Bechtolsheim et al [35]	2023	Prospective randomized study	dVSS	Not Serious	Serious	Not serious	Not serious	Moderate
Chowriappa et al [8]	2014	Randomized-controlled trial	HoST-AR	Not Serious	Serious	Not serious	Not serious	Moderate
Satava et al [38]	2020	Randomized-controlled trial	DVSS	Not Serious	Serious	Not serious	Not serious	Moderate
Whitehurst et al [37]	2015	Prospective randomized study	MdVT	Not Serious	Serious	Not serious	Not serious	Moderate

### Assessment of publication bias

**Fig. 9:**
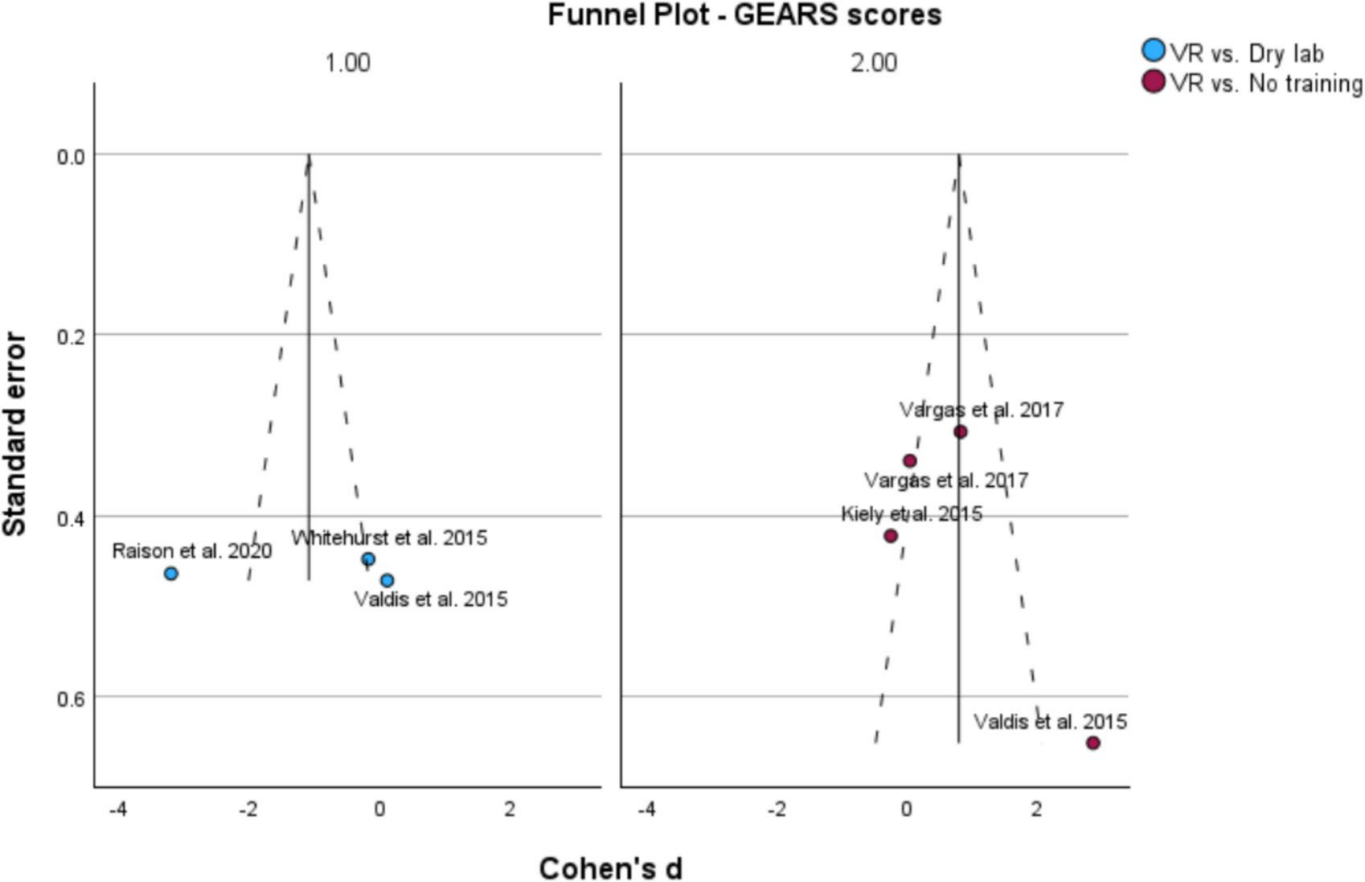
Funnel plot displaying the standard error of each study’s main effect size that compares the GEARS scores of participants

Funnel plots for each of the variable groups were generated to assess for publication bias. A symmetrical spread was found when standard effect was plotted against the main variables of time to complete and GEARS score; suggesting low publication bias (Fig. 9).

## Discussion

### Outcomes

Previous studies have shown that virtual reality simulators can be useful for preparing experienced surgeons for complicated surgeries preoperatively [39]. As well as training surgeons on laparoscopic procedures [40]. However, this article is the first to investigate if people who have never used a robotic surgical system can learn RAS skills using simulated exercises. This systematic review and meta-analysis use the most up to date randomized-controlled and prospective trials to determine if extended reality simulators can be used to effectively train robotic novices on how to use complex surgical robots. The summary of the meta-analyses completed in this study can be found in Table 5. Participants who received XR training completed tasks in significantly less time than participants with no training. The effect size of −0.95 is considered large, per Cohen’s d. The 95% confidence interval rested between −1.73 and −0.16, in favor of XR training. This means that surgeons trained with XR were able to complete tasks significantly quicker than those without training, showing adjustment to the machine and a level of confidence in performing the tasks given to them. The extended reality group also had higher GEARS scores than the no training group with an effect size of 0.75, which is a large effect on performance of those trained with extended reality. However, this value is not statistically significant due to having a *P* value of 0.23 which means that we are unable to determine whether the XR training significantly improves the surgical skill of a robotic novice over no additional training.

**Table 5:** Summary of meta-analysis results

Comparison	Cohen’s D	Confidence intervals	*P* value
Extended reality vs. No training Time to complete	−0.95	−1.73, -0.16	0.02
Extended reality vs. No training GEARS Score	0.75	−0.48, 2.05	0.23
Extended reality vs. Dry lab time to complete	0.65	−0.22, 1.52	0.14
Extended reality vs. Dry lab GEARS Score	−1.09	−3.17, 0.98	0.30

This review also compared how effectively XR simulators trained novice surgeons compared to the more commonly used training method of inanimate models (dry labs). Participants who received XR training completed tasks slower than participants with dry lab training. This effect size is considered moderate with Cohen’s d of 0.65. However, this result is not statistically significant with a *p* value of 0.14 and a 95% confidence interval that rests between −0.22 and 1.52, indicating that the results do not favor dry labs over XR. The GEARS scores of surgeons trained on dry labs are larger than those trained by XR with an effect size of 0.65, which is a moderate-effect size and was not found to be statistically significant due to having a P value of 0.30. Due to both results comparing the two training modalities not being statistically significant, we cannot conclude that one is a better training method than another.

GEARS scores have been shown to be a commonly used way to grade the performance of a surgery within a training setting [41]. However, due to the GEARS protocol having subjective judging from reviewers, there is still a level of variability and bias involved in using it as a metric for performance. Especially in a review, comparing GEARS scores between testing settings can be challenging due to the variation in reviewers, task being tested, or what the surgical task is being performed on. For example, while many of the studies used the surgical simulator as the final testing model, some used physical models like Vargas et al., 2017 using a porcine model. On top of this, the articles in this review include robotic novices ranging from medical students to attending physicians, leading to a wide variety in surgical experience outside of surgical robotics, and variation in surgical performance between studies. These variations in testing may have led to variation in participant’s GRADE scores and to poor statistical analysis using the meta-analysis model. All the assessment models used can be found in the summary of findings table.

Due to the variability of GEARS scores collected from the different studies, the authors also performed an independent sample *t* test to compare the means between groups as a more global comparison of surgical performance using the GEARS scores. The XR trained group had a significantly higher average GEARS score (16.73) compared to the no training group (13.91). The margin of GEARS indicates better technical skills acquisition via XR training. The effect size of GEARS scores between XR and No Training groups was strongly in favor of XR training (0.964, 95% CI 0.589 to 1.336), which is considered a large effect size. The results of the analysis are statistically significant with a *p* value of < 0.001, which points to the idea that XR simulators can be used as an effective way to train surgeons compared to just having the surgeons become familiar with the robot before use.

When comparing extended reality to inanimate dry lab models, the GEARS scores of the XR group (13.43) and dry lab group (14.08) are also inconclusive with an effect size of −0.093 and a *p* value of 0.34, suggesting no significant difference in the quality of performance of participants in both groups. The wide confidence interval for the GEARS score effect size between XR and Dry Lab training (−0.530 to 0.345) indicates a range from strong favor for Dry Lab to moderate favor for XR training. These results indicate that XR training and dry lab training are comparable training methods and lead to comparable results for surgeons.

Based on this analysis, we determined that extended reality training can be used as a model to improve robotic novices’ confidence in using robotic surgical devices. This is due to XR training improving TTC surgical tasks over participants with no training and having comparable results in both TTC and GEARS to the dry lab training. The GEARS scores being inconclusive in both studies, which is most likely due to the large variation in surgical experience between participants. While all participants were surgical novices, attending surgeons will have more competence in standard surgical procedures over medical students, leading to increased variability within the performance evaluation outside of the training itself. This means that it is inconclusive if the surgical performance is improved with the training, and more research with standardized participant surgical experience would be required for a more concrete answer. The non-statistical significance is also unlikely to be due to publication bias, due to the funnel plot test showing a low publication bias.

The differences between the dry lab training and extended reality training could be due to several factors as well. Examples include the simulator not being realistic enough, or some of the trials using inanimate models or virtual reality as their testing medium, giving one form of training an advantage over the other in the evaluation. However, with continued advances in virtual reality technology including better imaging and improved haptic feedback, the ability of surgical simulators to improve compared to dry lab simulators is present [42]. On top of the ability to continue to improve simulators, virtual reality offers some advantages over dry lab. With simulators, participants can retry a task as many times as they want, without needing to reset a physical model. As well as the ability to access a variety of different exercises created to simulate a variety of different scenarios, while a physical model might be limited in the number of variations a participant can practice. For example, the dVSS contains at least 23 basic simulation exercises, while a physical model may only be able to have a few exercises made at a time [11].

Finally, the cost of simulators is much less than using surgical robots to train surgeons. The average cost for a dVSS simulator is 127,000 dollars, while the cost of a full da Vinci surgical robot costs 1–2.5 million dollars alone, which does not include maintenance costs or operating room time. To train a surgeon using a da Vinci robot would cost a program significantly more than buying a trainer that requires less maintenance while also freeing a hospital’s surgical robot to be used for surgery instead of training. Due to this, dry labs are one of the most used training methods for novice surgeons right now. The development and improvement of surgical simulators should lead to more simulators being implemented into surgical training programs.

### Limitations

The largest challenge with this review is the unstandardized way participants were assessed after being trained. Most studies used different organ systems, models, or exercises to assess their surgeons, which lead to a wide variety of results, as each final task had a different standard of time to complete. The other risk of bias is the participant selection. Different studies used participants from a wide range of medical experience, as some studies used medical students, while others used attending surgeons. This led to major differences in the general skill level of the participants, even if they were all inexperienced with surgical robotics. Finally, while many studies used the same simulators, various training simulators with different durations of training were included in this review as well. This variation in training could lead to inconsistency in time and different GEARS results.

## Conclusion

RAS is particularly demanding to learn. However, through advances in virtual reality, augmented reality, and mixed reality, novice surgeons can practice these challenging surgeries without putting patients at risk. Many practice modalities have been created for this purpose, including animal models and inanimate models. However, extended reality simulators allow for an increase in variability in situation tasks and repeatability that these other models do not offer. This study is the first to compare surgical performance of different training modalities to extended reality post-training and supports the use of implementing extended reality into the training of surgical residents.

## Data Availability

The datasets and analysis scripts supporting this study are available from the corresponding author upon reasonable request.
